# Kinase Inhibitor Profile for Human Nek1, Nek6, and Nek7 and Analysis of the Structural Basis for Inhibitor Specificity

**DOI:** 10.3390/molecules20011176

**Published:** 2015-01-13

**Authors:** Eduardo Cruz Moraes, Gabriela Vaz Meirelles, Rodrigo Vargas Honorato, Tatiana de Arruda Campos Brasil de Souza, Edmarcia Elisa de Souza, Mario Tyago Murakami, Paulo Sergio Lopes de Oliveira, Jörg Kobarg

**Affiliations:** 1 LaboratórioNacional de Biociências, Centro Nacional de PesquisaemEnergia e Materiais, Campinas, 13083-970 SP, Brazil; E-Mails: eduardo.moraes@bioetanol.org.br (E.C.M.); gabriela.meirelles@lnbio.cnpem.br (G.V.M.); rodrigo.honorato@lnbio.cnpem.br (R.V.H.); edmarcia.souza@lnbio.cnpem.br (E.E.S.); mario.murakami@lnbio.cnpem.br (M.T.M.); paulo.oliveira@lnbio.cnpem.br (P.S.L.O.); 2 Programa de Pós-graduação em Biologia Funcional e Molecular, Departamento de Bioquímica e BiologiaTecidual, Instituto de Biologia, UniversidadeEstadual de Campinas, Campinas, 13083-862 SP, Brazil; 3 Instituto Carlos Chagas, FIOCRUZ, Curitiba, 81350-010 PR, Brazil; E-Mail: tatiana.brasil@gmail.com; 4 Departamento de Bioquímica e de Biologia Tecidual, Instituto de Biologia, Universidade Estadual de Campinas, Campinas, 13083-862 SP, Brazil; 5 Faculdade de Ciências Farmacêuticas, Universidade Estadual de Campinas, Campinas, 13083-859 SP, Brazil

**Keywords:** drug discovery, compound screening, kinase assays, molecular modeling, molecular docking, ATP-competitive inhibitors, non-competitive inhibition, Neks

## Abstract

Human Neks are a conserved protein kinase family related to cell cycle progression and cell division and are considered potential drug targets for the treatment of cancer and other pathologies. We screened the activation loop mutant kinases hNek1 and hNek2, wild-type hNek7, and five hNek6 variants in different activation/phosphorylation statesand compared them against 85 compounds using thermal shift denaturation. We identified three compounds with significant *T*_m_ shifts: JNK Inhibitor II for hNek1(Δ262-1258)-(T162A), Isogranulatimide for hNek6(S206A), andGSK-3 Inhibitor XIII for hNek7wt. Each one of these compounds was also validated by reducing the kinases activity by at least 25%. The binding sites for these compounds were identified by *in silico* docking at the ATP-binding site of the respective hNeks. Potential inhibitors were first screened by thermal shift assays, had their efficiency tested by a kinase assay, and were finally analyzed by molecular docking. Our findings corroborate the idea of ATP-competitive inhibition for hNek1 and hNek6 and suggest a novel non-competitive inhibition for hNek7 in regard to GSK-3 Inhibitor XIII. Our results demonstrate that our approach is useful for finding promising general and specific hNekscandidate inhibitors, which may also function as scaffolds to design more potent and selective inhibitors.

## 1. Introduction

Protein kinases (PKs) play an important role in the activation of biochemical pathways in eukaryotic cells. With over 500 described kinases encoded in the human genome, virtually every signal transduction process is wired through a phosphotransfer cascade. Therefore, the tight regulation of phosphorylation is crucial for cell growth and development, and this regulation relies on the proper regulation of kinase proteins. Protein kinase domains are found in ~2% of eukaryotic genes [1], which also reflects their importance. These proteins typically share a conserved arrangement of secondary structure elements into 12 subdomains that fold into a bi-lobed catalytic core structure with ATP binding in a deep cleft located between the lobes [2], and their homologous catalytic domain consists of ~250–300 amino acid residues [3]. PKs catalyze the transfer of the γ-phosphate of ATP to the hydroxyl group of serine, threonine or tyrosine of the substrate protein and are considered “molecular switches”, since they can adopt an “on” state [4,5,6,7].

NIMA-related kinases (Neks) are a conserved serine/threonine PK family related to cell cycle progression and cell division. These proteins share about 40% identity in the N-terminal catalytic domain to the NIMA (never in mitosis, gene A) protein of *Aspergillus nidulans*, which performs mitotic roles similar to the mammalian Neks [8,9]. Among these, Nek2 is the best studied, being described as a protein located in the centrosomes and kinetochores [10] that regulates the centrosome separation in the G2/M transition, thus being fundamental for the mitotic spindle formation [11]. Nek6 and Nek7 are the smallest proteins of the Nek family. Most of their structure is constituted of the catalytic domain, which is located in the C-terminal region [12]. Endogenous Nek6 is activated during mitosis and has its expression level increased during this process [13]. Overexpression of inactivated mutants of Nek6 and Nek7 shows similar phenotypes, with cells displaying high division rates, defects in the mitotic spindle, abnormal nuclei, and apoptosis [14,15]. These phenotypes are also observed by the iRNA silencing of Nek6 and Nek7 in Hela cells, leading to a cell cycle arrest in metaphase, with cells showing normal chromosome condensation but a disability to segregate them. Therefore, Nek6 and Nek7 play a crucial role in cell cycle progression to the anaphase [14,15,16]. Nek9 is also described to interact with Nek6 and Nek7, where the latter are activated by the phosphorylation of the former, which is activated during mitosis [13,17].

Moreover, several Neks are products of genes related to pathologies, such as diseases associated with defects in cell cycle progression and DNA repair mechanisms during interphase, especially carcinomas [9]. Mutation in Nek1 and Nek8 genes are related to polycystic kidney disease [18,19], Nek6 overexpression is observed in hepatocellular carcinomas [20], and studies also show that Nek6 and Nek1 are related to DNA damage checkpoints [21,22].

Due to these characteristics, Neks have emerged as potential targets for treatment of cancers and development of anti-cancer drugs. Hence, there is a great interest in the identification of potential inhibitors for these kinases. Some of these inhibitors were experimentally proposed for human Nek2 [23,24,25], others were only evaluated by *in silico* techniques for hNek6 [26], though for the majority of Neks these studies remain elusive, in part due to the lack of a crystal structure, which isnow available only for hNek2 [27,28], hNek7 [29], and hNek1. In this context, a number of recent successful drugs have emerged from a structure-based research approach [30], and most of the efforts to develop low molecular-weight inhibitors that have entered clinical programs are focused on ATP-competitive compounds.

Here we present the evaluation of the efficiency of an ATP-competitive compounds library by the use of three procedures: thermal shift assays, kinase activity assays and molecular docking. Thermal shift assays allow quantifying the stability of the protein-ligand interaction by the protein unfolding in an increasing temperature range [31]. Kinase activity assays evaluate the increase or decrease of enzyme activity in the presence of ligands by the phosphorylation rate of a certain substrate. Molecular docking provides *in silico* evaluation of the protein-ligand interaction region and affinity calculated using a scoring function based on an approximate force field [32]. Thereby, potential inhibitors were first screened by thermal shift assays, had their efficiency tested by a kinase inhibition assay and had their conformation and placement predicted by molecular docking.

## 2. Results and Discussion

### 2.1. Screening of ATP-Competitive Inhibitors for Recombinant Human Neks 1, 2, 6, and 7 by Thermal Shift Assay

Aiming to find possible inhibitors for hNeks 1, 6 and 7 in a drug design approach, we performed a screen using Inhibitor Select™ 96-Well Protein Kinase Inhibitor Library II (Calbiochem), containing 80 inhibitors targeting mostly Ser/Thr kinases, and five other compounds (AMP, ADP, ATP, ATP-g-S, and SU11652) (Supplementary File). In a previous work using the thermal stability shift assay, 156 validated kinase inhibitors were screened against 60 human Ser/Thr kinases, including recombinant hNek2 and hNek6, but no significant *T*_m_ shift (Δ*T*_m_> 4.0 °C) was detected for hNek6 [33]. Here we screened 85 compounds against recombinant hNek1(Δ262-1258)-(T162A), hNek7wt and five recombinant hNek6 variants—6xHis-hNek6wt, 6xHis-hNek6(S206A), 6xHis-hNek6(Δ1-44), 6xHis-hNek6wtD and 6xHis-hNek6(S206A)D—and used hNek2(Δ272-445)-(T175A) as a control, since it had already been used in the previous screen by Fedorov *et al.* [33], in order to search for potential inhibitors. We were also interested in observing whether the hNek6 activation/phosphorylation status might interfere with its stability in the presence of different compounds and whether this characteristic could influence the search and/or development of novel inhibitors for this kinase. In this context, thermal shift assays for the five recombinant hNek6 variants were described to reveal a slightly higher stability for wild-type hNek6 compared to the activation loop mutant [12].

From this screen, we were able to retrieve one compound with significant *T*_m_ shift for hNek1(Δ262-1258)-(T162A), JNK Inhibitor II, and one for hNek6(S206A), Isogranulatimide (Table 1). On the other hand, hNek7wt and our control, hNek2(Δ272-445)-(T175A), retrieved 10 and 5 hit compounds, respectively, with significant *T*_m_ shifts: Cdk1/2 Inhibitor III, GSK-3 Inhibitor XIII, PD 169316, JNK Inhibitor V, MK2a Inhibitor, Indirubin-3'-monoxime, Isogranulatimide, K-252a, Indirubin Derivative E804and SB 218078for hNek7wt; and Aminopurvalanol A, NF-kB Activation Inhibitor, JNK Inhibitor II, K-252a and SU11652 for hNek2(Δ272-445)-(T175A) (Table 1). The compound SU11652, retrieved here for hNek2(Δ272-445)-(T175A), had also been identified for this kinase with a significant *T*_m_ shift (Δ*T*_m_ = 5.1 °C) in the previous screen by Fedorov *et al.* [33], contributing to validate our assays.

**Table 1:** Summary of recombinant hNeks 1, 2, 6 and 7 thermal shift inhibitor screen showing only compounds withΔ*T*_m_ ≥ 2 °C. Names of compounds showing a significant thermal shift (Δ*T*_m_ ≥ 4 °C) are depicted in bold.

Nek	CAS Number	Compound Description	Δ *T_m_* (°C)
**Nek1(Δ262-1258)-(T162A)**	667463-62-9	GSK-3 Inhibitor IX	3.5
**Nek1(Δ262-1258)-(T162A)**	444723-13-1	Cdk2 Inhibitor IV, NU6140	3.1
**Nek1(Δ262-1258)-(T162A)**	879127-16-9	Aurora Kinase Inhibitor III	3.6
**Nek1(Δ262-1258)-(T162A)**	443798-55-8	Cdk1/2 Inhibitor III	2.6
**Nek1(Δ262-1258)-(T162A)**	220792-57-4	Aminopurvalanol A	2.5
**Nek1(Δ262-1258)-(T162A)**	4129-56-6	**JNK Inhibitor II**	**4.0**
**Nek2(Δ272-445)-(T175A)**	220792-57-4	**Aminopurvalanol A**	**4.0**
**Nek2(Δ272-445)-(T175A)**	866405-64-3	AMPK Inhibitor, Compound C	2.5
**Nek2(Δ272-445)-(T175A)**	326914-10-7	**SU11652**	**9.6**
**Nek2(Δ272-445)-(T175A)**	4129-56-6	**JNK Inhibitor II**	**4.8**
**Nek2(Δ272-445)-(T175A)**	97161-97-2	**K-252a, Nocardiopsis sp.**	**5.4**
**Nek2(Δ272-445)-(T175A)**	522629-08-9	MNK1 Inhibitor	2.1
**Nek2(Δ272-445)-(T175A)**	545380-34-5	**NF-kB Activation Inhibitor**	**4.2**
**Nek2(Δ272-445)-(T175A)**	601514-19-6	GSK3b Inhibitor XII, TWS119	2.3
**Nek6(S206A)**	62996-74-1	Staurosporine, Streptomyces sp.	2.1
**Nek6(S206A)**	852527-97-0	Alsterpaullone, 2-Cyanoethyl	2.5
**Nek6(S206A)**	244148-46-7	**Isogranulatimide**	**6.5**
**Nek7wt**	345987-15-7	**JNK Inhibitor V**	**4.9**
**Nek7wt**	443798-55-8	**Cdk1/2 Inhibitor III**	**4.2**
**Nek7wt**	135897-06-2	**SB 218078**	**11.5**
**Nek7wt**	152121-53-4	**PD 169316**	**4.4**
**Nek7wt**	62996-74-1	Staurosporine, Streptomyces sp.	3.2
**Nek7wt**	97161-97-2	**K-252a, Nocardiopsis sp.**	**8.8**
**Nek7wt**	244148-46-7	**Isogranulatimide**	**8.3**
**Nek7wt**	404828-08-6	**GSK-3 Inhibitor XIII**	**4.3**
**Nek7wt**	220792-57-4	Aminopurvalanol A	3.0
**Nek7wt**	41179-33-3	**MK2a Inhibitor**	**5.3**
**Nek7wt**	854171-35-0	**Indirubin Derivative E804**	**9.7**
**Nek7wt**	160807-49-8	**Indirubin-3'-monoxime**	**7.9**
**Nek7wt**	487021-52-3	GSK-3b Inhibitor VIII	3.3
**Nek7wt**	4129-56-6	JNK Inhibitor II	2.6

Our results also show that one compound, Isogranulatimide, produced a significant *T*_m_ shift for hNek6(S206A), but not for the other variants (Table 2). Moreover, the activation loop mutant, dephosphorylated or not, showed a higher number of cases in which a compound produced a *T*_m_ shift of at least 2 °C—three cases for hNek6(S206A) and ten cases for hNek6(S206A)D—compared to the wild-type hNek6—no cases for hNek6wt, hNek6wtD or 6xHis-hNek6(Δ1-44) (Table 2). These data together suggest that a partially activated hNek6 kinase, without being phosphorylated at its activation loop, is a better target for inhibitor stabilization than an activated more phosphorylated kinase.

**Table 2:** Summary of hNek6 variants thermal shift inhibitor screen. A thermal shift of at least 2 °C (Δ*T*_m_ ≥ 2 °C) is depicted in italics and a significant thermal shift (Δ*T*_m_ > 4 °C) is depicted in bold. (D: dephosphorylated).

CompoundDescription	Nek6wt	Nek6wtD	Nek6(S206A)	Nek6(S206A)D	Nek6(Δ1-44)
Alsterpaullone	0.0	0.1	1.4	*2.9*	−0.5
Alsterpaullone, 2-Cyanoethyl	0.0	−0.1	*2.5*	*2.5*	−0.2
Aminopurvalanol A	0.3	−1.8	0.8	*_*	−0.2
Aminopyrazole1	−0.4	0.1	1.0	*2.4*	0.0
AR-A014418	0.3	−0.5	1.4	*2.6*	−0.2
Indolinone1	_	−0.4	−0.2	*2.1*	−0.5
Indolocarbazole1	−0.3	−0.1	1.7	0.7	−1.3
Isogranulatimide	1.2	0.6	**6.5**	*2.3*	−0.7
JNJ-7706621	0.0	0.6	0.7	*2.1*	−0.7
Pyrazolanthrone	0.3	−1.1	1.8	*2.3*	0.1
Staurosporine	0.3	−0.7	*2.1*	*2.3*	_
TPCA-1	0.0	−0.7	0.9	*2.0*	−1.7

In this context, the Isogranulatimide compound was identified as a G_2_ DNA damage checkpoint inhibitor containing a unique indole/maleimide/imidazole skeleton in a phenotypic cell-based screen [34], and *in vitro* kinase assays showed that it inhibits Chk1 (IC_50_ = 0.1 μM) and GSK-3 beta (IC_50_ = 0.5 µM) [35]. Human Nek6 hasrecently been described to be phosphorylated upon exposure to Ionizing radiation or UV irradiation through the DNA damage checkpoint *in vivo*, probably by the checkpoint kinases Chk1 and Chk2, which is suggested to be required for proper cell cycle arrest in the G_2_/M phase upon DNA damage [22]. Therefore, since hNek6 is involved in the G_2_/M phase cell cycle arrest through DNA damage-induced phosphorylation and is also a target of Isogranulatimide, besides Chk1 itself, hNek6 may be a promising candidate for modulating checkpoint responses in tumors for therapeutic benefit.

### 2.2. Kinase Assays of Selected Candidate Compounds for Recombinant Human Neks 1, 6, and 7

In order to confirm whether the retrieved compounds from the thermal shift assays could also inhibit the kinases activities, *in vitro* kinase assays were performed. As expected, JNK Inhibitor II compound retrieved in our screen for hNek1(Δ262-1258)-(T162A) with a significant *T*_m_ shift (Δ*T*_m_ = 4.0 °C) was able to reduce its activity in almost 30% (to 71.5% ± 0.1%) at a 50 µM concentration (Table 3). As also expected for hNek6(S206A), the Isogranulatimide compound showing a significant *T*_m_ shift (Δ*T*_m_ = 6.5 °C) was able to reduce its activity to 74.9% ± 10.4% at 0.625 μM. Moreover, regarding hNek7wt, one compound, GSK-3 Inhibitor XIII, with a significant *T*_m_ shift (Δ*T*_m_ = 4.3 °C) reduced hNek7 activity to 46.3% ± 2.9% (1.25 µM) and 43.3% ± 10.1% (0.312 µM). Although not showing *T*_m_ shifts above 4.0 °C, two other compounds were also effective in reducing hNek7 activity: Aminopurvalanol A (Δ*T*_m_ = 3.0 °C) and GSK-3b Inhibitor VIII (Δ*T*_m_ = 3.3 °C). Aminopurvalanol A reduced hNek7 activity up to 35.9% ± 3.3% (0.625 µM), while GSK-3b Inhibitor VIII reduced it up to 44.3% ± 3.3% (0.312 µM) (Table 3).

### 2.3. Molecular Docking of Selected Inhibitors for Recombinant Human Neks 1, 6, and 7

**Figure 1:**
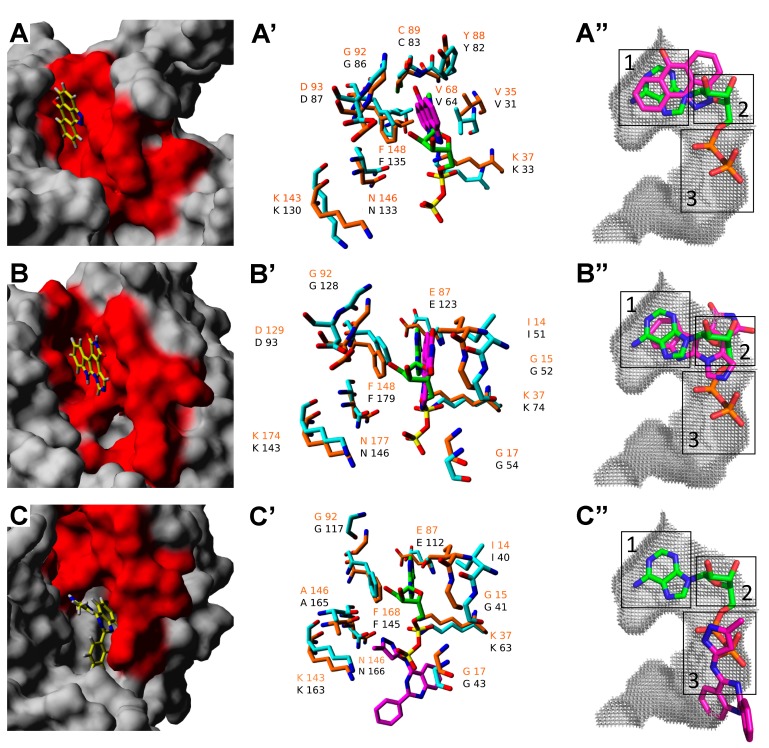
Representationofbestcandidate compounds complexed to recombinant hNeks 1, 6, and 7 in comparison withhNek2 in the ATP-binding site. (**A–C**) Three-dimensional representation of the top docking position for each compound-target complex in the ATP-binding site (red). (**A’–C’**) Three-dimensional representation of the interactions between each target protein (cyan) and candidate compound (magenta) in comparison to our reference hNek2 (PDB: 2W5A) (orange) boundto ADP (green). Amino acid residue labels are depicted in orange for the reference hNek2 and in black for the other hNeks. (**A”–C”**) Three-dimensional representation of the preferable area occupied by candidate compounds (magenta) relative to ADP (green) in the ATP-binding site cavity (grey) of our reference hNek2 (PDB: 2W5A) identified by KV Finder. The binding subsites are depicted as follows: (1) adenine region; (2) ribose region; and (3) phosphate groups region. (A) hNek1(Δ262-1258)-(T162A) bound to JNK Inhibitor II; (B) hNek6(S206A) bound to Isogranulatimide; (C) hNek7wt bound to GSK-3 Inhibitor XIII.

Selected candidate inhibitors (Table 3) were subjected to global docking analysis and had their energy and conformation analyzed. The ATP-binding site was identified on each target-ligand complex based on the molecular models of hNek1(Δ262-1258)-(T162A) and hNek6(S206A) and on the crystal structure of hNek7 (PDB: 2WQN) [29]. All docking conformations were ranked based on their energy values, and it was observed that the top hit conformations (lowest binding energy) of each complex were located in the ATP-binding site (Figure 1A–C). Total predicted ligand-protein binding energies are depicted in Table 3.

The ATP-binding site in hNeks is characterized by a pocket predominantly positively charged responsible for anchoring the polar tail of ATP. This site is very conserved among Neks, and in our reference hNek2 (PDB: 2W5A) it is structurally constituted by the following residues: I^14^, G^15^, T^16^, G^17^, S^18^, Y^19^, G^20^, R^21^, C^22^, V^35^, K^37^, V^68^, M^86^, E^87^, Y^88^, C^89^, E^90^, G^92^, D^93^, S^96^, K^143^, A^145^, N^146^, F^148^, D^159^, F^160^, and R^164^. The ATP-binding region signature considering only the primary sequence analysis in ScanProsite [36] for all 11 human Neks consists of about 23 amino acid residues between I14 and K37 (hNek2 numbering) [12]. Interestingly, 10 interactions (about 40%) in hNeks 1,6 and 7 bound to their respective candidate compounds in a corresponding way as  the interactions that occurr in the ATP-binding pocket of hNek2 (PDB: 2W5A) with ADP (Figure 1A’–C’).

**Table 3:** Summary of compounds with thermal shifts of at least 3 °C (Δ*T*_m_ ≥ 3 °C), total predicted ligand-protein binding energies in ATP-binding site and percent phosphorylation activity up to 75% for recombinant hNeks 1, 6, and 7. The percent phosphorylation is according to a compound final concentration of 2×, 1×, and 0.5× the ATP K_m[apparent]_ concentration of each kinase. The ATP K_m[apparent]_ concentrations found for Neks are as follows: 0.625 µM for hNek6(S206A) and hNek7wt, and 25 µM for hNek1(Δ262-1258)-(T162A). Only percentage of phosphorylation values with standard errors < 20 are depicted in the table. Names of compounds corresponding to Δ*T*_m_ ≥ 4.0 °C and phosphorylation ≤ 75% are depicted in bold and were selected as the best candidates for hNeks.

Nek	CAS Number	Compound Description	Δ*T_m_* (°C)	Docked/Predicted Binding Energy (kcal/mol)	Phosphorylation (%) 2× ^b^	**Phosphorylation (%) 1× ^b^**	**Phosphorylation (%) 0.5× ^b^**
**Nek1(Δ262-1258)-(T162A)**	4129-56-6	**JNK Inhibitor II**	4.0	−7.345	71.5 ± 0.1	87.3 ± 18.3	-
**Nek6(S206A)**	244148-46-7	**Isogranulatimide ^a^**	6.5	−8.917	-	74.9 ± 10.4	100.2 ± 1.3
**Nek7wt**	220792-57-4	Aminopurvalanol A	3.0	−8.626	41.0 ± 13.5	35.9 ± 3.3	64.4 ± 3.4
**Nek7wt**	487021-52-3	GSK-3b Inhibitor VIII	3.3	−7.572	58.1 ± 3.2	47.5 ± 4.4	44.3 ± 3.3
**Nek7wt**	404828-08-6	**GSK-3 Inhibitor XIII**	4.3	−8.629	46.3 ± 2.9	-	43.3 ± 10.1

^a^: Isogranulatimide was selected for hNek6(S206A), although corresponding to a minimum percentage of phosphorylation of 74.9 ± 10.4; ^b^: S.E. of two measurements.

**Figure 2:**
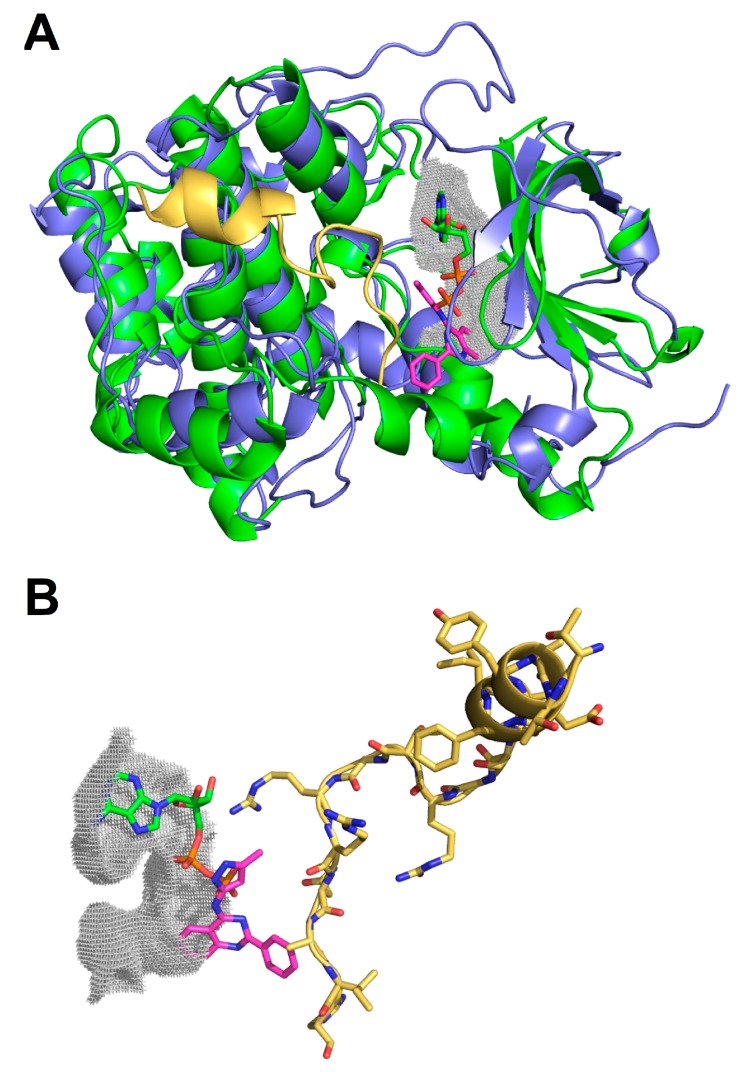
Representation of PKI complexed to PKAC-alpha and the relative position of GSK-3 Inhibitor XIII in the ATP and substrate-binding sites of hNek2. (**A**) Structural alignment between PKAC-alpha (PDB: 1FMO) (blue) and hNek2 (PDB: 2W5A) (green), both displayed in cartoon representation, with a root mean square deviation of 2.010 Å. The PKI peptide inhibitor is depicted in yellow whereas the GSK-3 Inhibitor XIII compound is shown in magenta alongside the ADP molecule (green) inside the ATP-binding site; (**B**) Detailed representation of the most favorable conformation adopted by GSK-3 Inhibitor XIII (magenta) that could block the access to the substrate-binding site.

Moreover, the same cavity occupied by ADP in the crystal structure of hNek2 (PDB: 2W5A) could also be occupied by the candidate compounds identified for hNek1(Δ262-1258)-(T162A), hNek6(S206A) and hNek7wt, where JNK Inhibitor II preferentially occupies the adenine region, Isogranulatimide extends to the ribose region, and GSK-3 Inhibitor XIII occupies the phosphate groups region and beyond (Figure 1A”–C”). Surprisingly, from the three candidate compounds, two behave as expected ATP-competitive inhibitors, but one, GSK-3 Inhibitor XIII, is probably a non-ATP-competitive inhibitor of hNek7, since our cavity analysis suggests it also occupies a region corresponding to the substrate-binding site (Figure 2).

In a recent review on approaches to discover non-ATP site kinase inhibitors by Gravin and Saiah (2012), Type III kinase inhibitors were described as small molecule inhibitors that bind in a pocket adjacent to the ATP site, such as the MEK inhibitor CI-1040, while Type V were described as a class of bivalent kinase inhibitors consisting ofa peptide analog to the substrate attached covalently to an ATP competitive small molecule [37]. Therefore, GSK-3 Inhibitor XIII has characteristics of a Type III, but also Type V kinase inhibitor, since it is a small molecule inhibitor that binds in a pocket that extends from the ATP to the substrate site. This is in agreement with the kinase assays performed for hNek7, particularly for hNek7 bound to GSK-3 Inhibitor XIII, where the percentage of phosphorylation activity did not increase with the decreasing inhibitor concentration relative to the ATP concentration. In this case, a small concentration of the compound is sufficient to inhibit the kinase activity even if a relative higher amount of ATP is present, since the inhibitor is unlikely to compete with ATP for the same binding site in hNek7, but is most probably interfering with the peptide/substrate binding, contrary to hNek1 and 6 candidate inhibitors (Table 3). Moreover, our cavity analysis using KVFinder[38] suggests that there is a difference in the predicted binding regions of hNeks regarding their activation/phosphorylation status: the candidate compounds retrieved for the activation loop mutant hNeks occupy only the adenine and ribose-binding regions (Figure 1A”,B”) in contrast to the candidate compound retrieved for the wild-type hNek, which occupies mainly the phosphate groups-binding region extending to the substrate-binding site (Figure 1C”).

**Figure 3:**
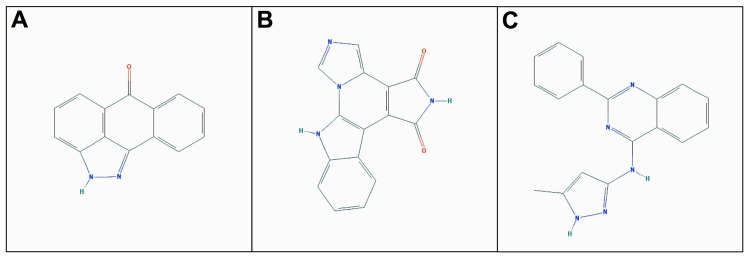
Two dimensional structure of the best candidate compounds. (**A**) JNK Inhibitor II; (**B**) Isogranulatimide; (**C**) GSK-3 Inhibitor XIII.

Hence, from an initial set of 85 compounds, three were identified as promising inhibitors for hNeks 1, 6, and 7 (their 2D structures are depicted in Figure 3). Though, since these are commercial compounds also described to inhibit JNK, Cdk1/2, and GSK-3, further cell-based assays will be important to validate their activity and specificity towards hNeks. All these findings, particularly the one regarding the hNek7 candidate, which probably interferes with substrate binding, should be taken into account in order to design more potent and selective inhibitors for hNeks.

## 3. Materials and Methods

### 3.1. Plasmid Constructions

To express truncated hNek1 corresponding to its kinase domain only fused to a 6xHis tag, a specific primer set was used, 5'-CGGAATTCCATATGGAGAAGTATGTTAGACTAC-3' and 5'-CGGGATCCTTAGCGTTTGGCTATAAAACCTTTCTC-3', to amplify the sequence from a full-length hNek1 cloned into pCMV5-FLAG kindly provided by Dr. Guido Lenz (Departamento de Biofísica, Instituto de Biociências, UFRGS, Porto Alegre, RS, Brazil). The resulting PCR-product was inserted into NdeI and BamHI restriction sites of the modified bacterial expression vector pET28a-TEV (Novagen/EMD Biosciences). Plasmids encoding hNek6 had previously been described by Meirelles *et al.* [39] and those encoding Nek7 were constructed in the same fashion. The orientation, frame, and sequence correctness of each DNA insert were confirmed by automated DNA sequencing.

### 3.2. Site-Directed Mutagenesis

The hNek1 and hNek6 activation loop mutations T162A and S206A, respectively, were introduced by PCR-based mutagenesis according to Meirelles *et al*. [39]. Mutation was confirmed by DNA sequencing. The truncated activation loopmutant hNek2corresponding to its kinase domain only fused to a 6×His tag—hNek2(Δ272-445)-(T175A)—cloned into pET22b (Novagen) was kindly provided by Dr. Stefan Knapp (Section of Structural Biology, Institute of Cancer Research, Oxford, UK). The activation loop mutant constructs of hNek1, 2, and 6 lead to a higher protein expression and lower toxicity, and also to an increased stability in the case of hNek1 and 2, besides enabling to study the influence of their activation/phosphorylation status in the search and/or development of novel inhibitors.

### 3.3. Protein Expression, Purification, and Dephosphorylation

Soluble truncated mutant hNek1—hNek1(Δ262-1258)-(T162A)—fused to a 6xHis tag was expressed in BL21 (DE3/pRARE) cells at 18 °C using 1.0 mM isopropyl 1-thio-β-d-galactopyranoside (IPTG) for 4 h, in Terrific Broth medium. Soluble truncated mutant hNek2—hNek2(Δ272-445)-(T175A) was expressed and purified according to Rellos *et al*. [27]. Soluble full-length wild-type hNek7—hNek7wt—fused to a 6xHis tag was expressed in *E. coli* BL21 (DE3/pRARE) or BL21 (DE3) cells. The cells were induced for 4 h using 1 mM of isopropyl-β-d-thio-galactoside (IPTG) at 28 °C. Induced cells were harvested and lysed by sonication in extraction buffer (50 mM HEPES pH 7.5; 5 mM sodium phosphate, 300 mM NaCl, 5% glycerol) plus 1 mM PMSF and 625 µg/mL lysozyme. The cell lysates were separated by centrifugation at 16,000× *g* for 10 min at 4 °C in order to obtain the supernatant. Cleared fraction of 6xHis-hNek7 obtained by lysis was purified by affinity liquid chromatography using HiTrap Chelating affinity chromatography column (GE Healthcare) and eluted with an imidazole gradient (1 to 100 mM) in extraction buffer. Soluble full-length hNek6 wild-type—6xHis-hNek6wt—and mutant—6xHis-hNek6(S206A)—or truncated hNek6 wild-type kinase domain—6xHis-hNek6(Δ1-44)—fused to a 6xHis tag were expressed and purified according to Meirelles *et al*. [39]. Dephosphorylated wild-type and mutant hNek6 were obtained according to Meirelles *et al*. [12].

### 3.4. Thermal Shift Assays

Thermal shift assays were performed according to Meirelles *et al.* [12]. Proteins were buffered in 10 mM HEPES pH 7.5, 150 mM NaCl and assayed at a final concentration of 2.0 µM in 25 µL volume in the presence of SYPRO-Orange (Molecular Probes, Eugene, OR, USA) at a dilution of 1 in 1000. OriginPro 8 software was used to fit data to the Boltzmann equation and *T*_m_ values were calculated by determination of the maximum of the first derivative. In the kinase inhibitor screen, the observed temperature shifts, Δ*T*_m_, for each inhibitor were recorded as the difference between the transition midpoints of sample and reference wells containing protein without inhibitor in the same plate. Observed temperature shifts above 4.0 °C were considered significant shifts, according to the work by Fedorov *et al*. [33].

### 3.5. Kinase Assays

Kinase assays were performed using LANCE^®^*Ultra* TR-FRET kinase assay protocol (PerkinElmer Life and Analytical Sciences, Shelton, CT, USA). IC_50_ values were determined by using a kinase concentration that phosphorylated 20%–50% of the ULight™-labeled p70S6K (Thr389) Peptide substrate (PerkinElmer Life and Analytical Sciences) at the ATP K_m[apparent]_. Kinase and ATP concentrations that matched these characteristics had been obtained in a previous assay, using a kinase concentration range between 2.5 nM and 640 nM, and an ATP concentration range between 0 and 25 µM. Inhibitor final concentration was 2×, 1× and 0.5× the ATP K_m[apparent]_ concentration found for each kinase: 0.625 µM for hNek6(S206A) and hNek7wt, and 25 µM for hNek1(Δ262-1258)-(T162A).

### 3.6. Homology Molecular Modeling

Homology models for hNek1(Δ262-1258)-(T162A) and hNek6(S206A) were obtained based on the protein structure of hNek1 (PDB: 4APC) (unpublished) and hNek7 (PDB: 2WQN) [29], respectively, in which both the ATP and substrate sites were accessible, using YASARA (http://www.yasara.org). The alignment with the target sequence is improved using sequence-based profiles obtained from related Uniprot sequences (hNek1: Q96PY6-1; hNek6: Q9HC98-1; and hNek7: Q8TDX7-1) along with secondary structure prediction, to ensure high model quality.

### 3.7. Molecular Docking

Compounds showing the most significant Δ*T*_m_ (Δ*T*_m_ ≥ 4.0 °C) and increased inhibitory activity (percent phosphorylation ≤ 75%) were selected as best candidates and used as ligands for molecular docking against Neks. JNK Inhibitor II (Δ*T*_m_ = 4.0 °C, percent phosphorylation = 71.5 ± 0.1) and Isogranulatimide (Δ*T*_m_ = 6.5 °C, percent phosphorylation = 74.9 ± 10.4) were used considering hNek1(Δ262-1258)-(T162A) and hNek6(S206A) molecular models as targets, respectively. GSK-3 Inhibitor XIII (Δ*T*_m_ = 4.3 °C, percent phosphorylation = 46.3 ± 2.9) was used considering the hNek7 crystal structure as the molecular target. Compound structures were obtained from the commercial SDF catalog (Calbiochem) and submitted to energetic parametrization and minimization on YASARA (http://www.yasara.org). Global molecular docking simulations were conducted using Autodock VINA [40] with 25 runs and 5 Å clustering RMSD.

## 4. Conclusions

We screened the activation loop mutant kinases hNek1 and hNek2, wild-type hNek7 and five hNek6 variants, with different activation/phosphorylation states, and compared them against 85 compounds using thermal shift denaturation and identified one compound with significant *T*_m_ shift for hNek1(Δ262-1258)-(T162A), JNK Inhibitor II, and ten hit compounds for hNek7wt. Of these compounds, JNK Inhibitor II was validated by reducing hNek1(Δ262-1258)-(T162A) activity to 71.5% ± 0.1% at a 50 µM concentration, while GSK-3 Inhibitor XIII was validated by reducing hNek7wt activity to 46.3% ± 2.9%, at a 1.25 µM concentration. JNK Inhibitor II was also predicted to bind to the ATP-binding site of hNek1(Δ262-1258)-(T162A) as an ATP-competitive inhibitor, in contrast to GSK-3 Inhibitor XIII, which showed a novel possible non-ATP-competitive inhibition of hNek7, but a most probable peptide/substrate-competitive inhibition.

We also found that mutant hNek6, without the activation loop conserved phosphorylation, is a better target for inhibitor stabilization than an activated more phosphorylated hNek6 kinase. From this experiment, we retrieved one compound, Isogranulatimide, which produced a significant *T*_m_shift for hNek6(S206A), but not for the other variants, which was later confirmed to reduce hNek6(S206A) activity to 74.9% ± 10.4% at 0.625 µM and to bind to its ATP-binding site.

Further functional experiments in living cells are required to validate this findings, and structural studies with atomic resolution will be important to characterize the mechanistic association of hNek1(Δ262-1258)-(T162A), hNek6(S206A), and wild-type hNek7 with these promising compounds. Furhtermore, they may also function as scaffolds to design more potent and selective inhibitors for hNeks.

## Supplementary Materials

Supplementary materials can be accessed at: http://www.mdpi.com/1420-3049/20/01/1176/s1.
