# Supplementary material for: Kinase Inhibitor Profile for Human Nek1, Nek6, and Nek7 and Analysis of the Structural Basis for Inhibitor Specificity
Source: Molecules. 2015 Jan 13;20(1):1176–91. doi: 10.3390/molecules20011176 (PMC6272266; doi:10.3390/molecules20011176)
Supplement: Supplementary file 1 [file molecules-20-01176-s001.pdf]

Supplementary Materials

Table S1. List of compounds used in the thermal shift screenings. Note: The following information can be accessed at PubChem database [1]: http://pubchem.ncbi.nlm.nih.gov.

Description	CAS Number	Structure	PubChem Compound ID
**KN-62**	**127191-97-3**	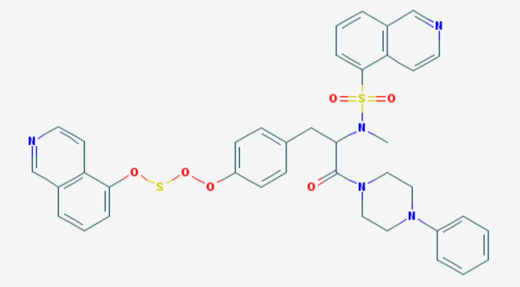	**16760529**
**ATM Kinase Inhibitor**	**587871-26-9**	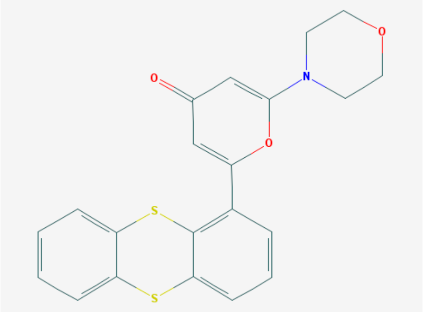	**5278396**
**ATM/ATR Kinase Inhibitor**	**905973-89-9**	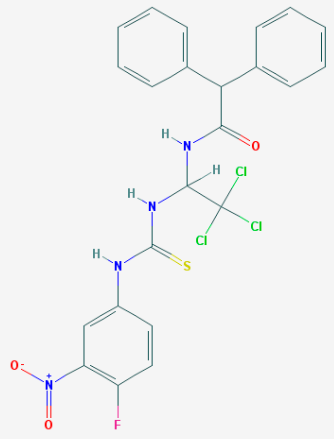	**6605258**
**Alsterpaullone**	**237430-03-4**	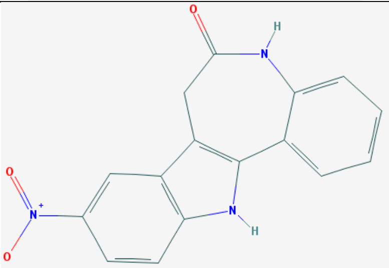	**5005498**
**Alsterpaullone, 2-Cyanoethyl**	**852527-97-0**	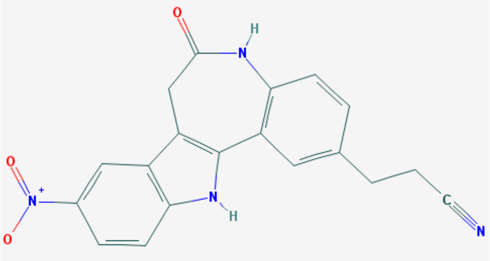	**16760286**

Table S1. *Cont.*

Description	CAS Number	Structure	PubChem Compound ID
Aloisine A, RP107	496864-16-5	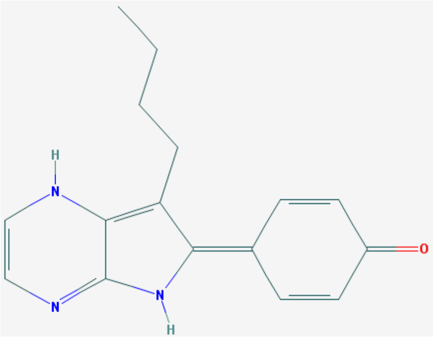	5326843
Aloisine, RP106	496864-15-4	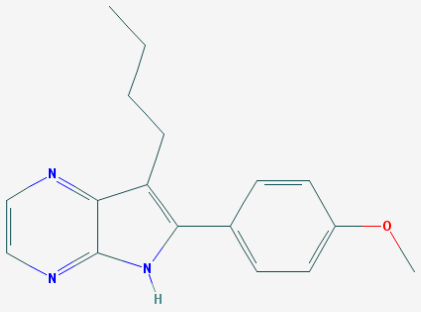	3641059
Aminopurvalanol A	220792-57-4	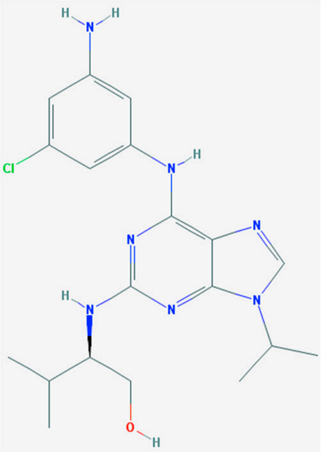	6604931
AMPK Inhibitor, Compound C	866405-64-3	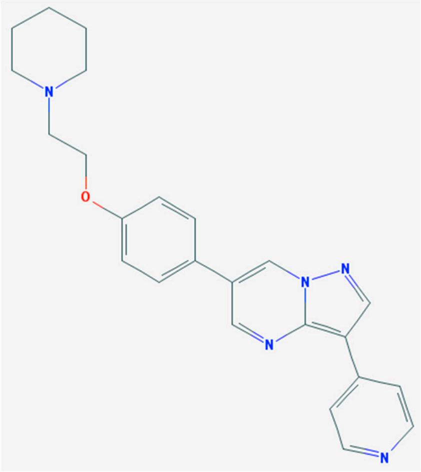	11524144

Table S1. *Cont.*

Description	CAS Number	Structure	PubChem Compound ID
Aurora Kinase Inhibitor III	879127-16-9	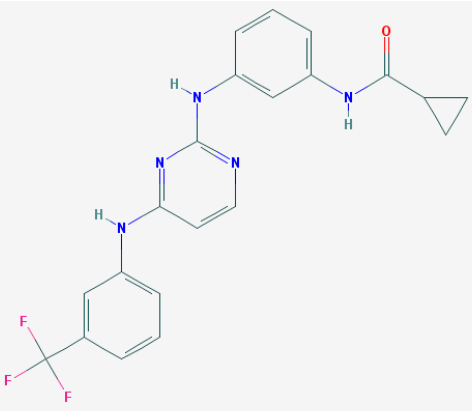	9549303
Aurora Kinase/Cdk Inhibitor	443797-96-4	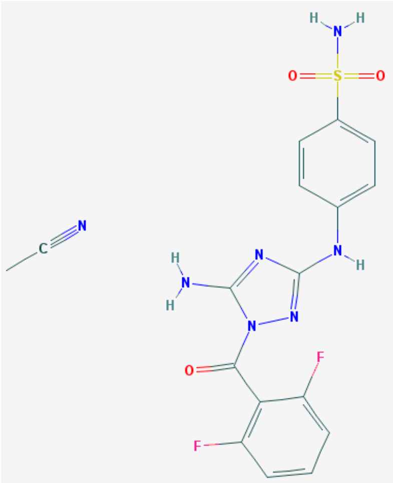	16760303
Indirubin-3′-monoxime	160807-49-8	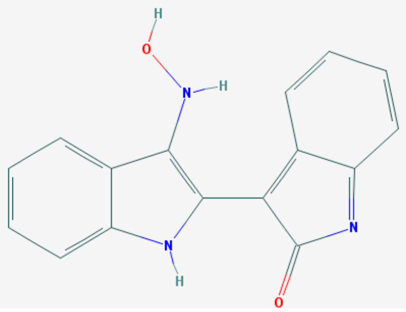	5326739
BAY 11-7082	19542-67-7	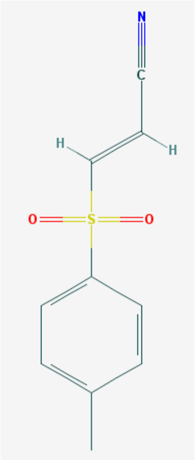	5353431

Table S1. *Cont.*

Description	CAS Number	Structure	PubChem Compound ID
**Bohemine**	**189232-42-6**	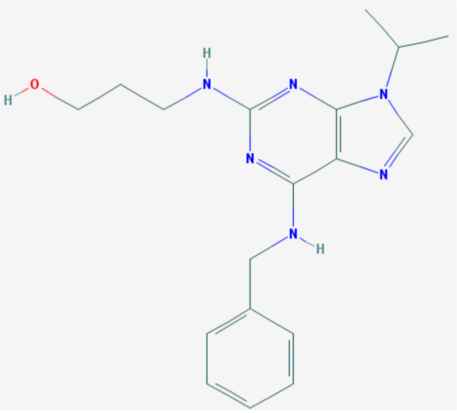	**2422**
**Cdk1 Inhibitor**	**220749-41-7**	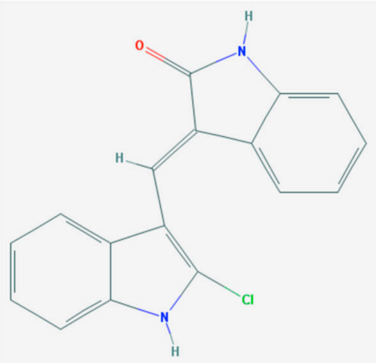	**5472558**
**Cdk1 Inhibitor, CGP74514A**	**190654-01-4**	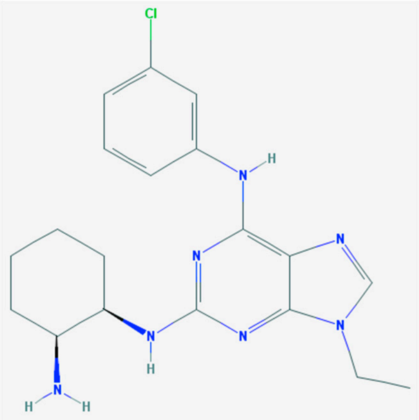	**2794188**
**Cdk1/2 Inhibitor III**	**443798-55-8**	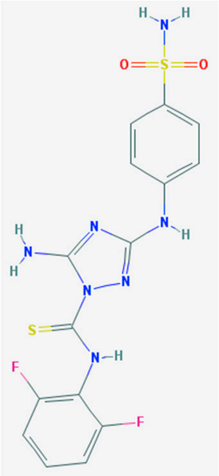	**5330812**

Table S1. *Cont.*

Description	CAS Number	Structure	PubChem Compound ID
Cdk1/5 Inhibitor	40254-90-8	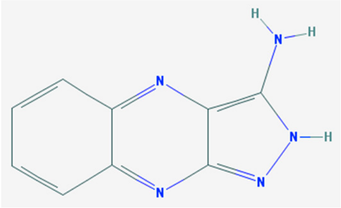	438981
Casein Kinase I Inhibitor, D4476	301836-43-1	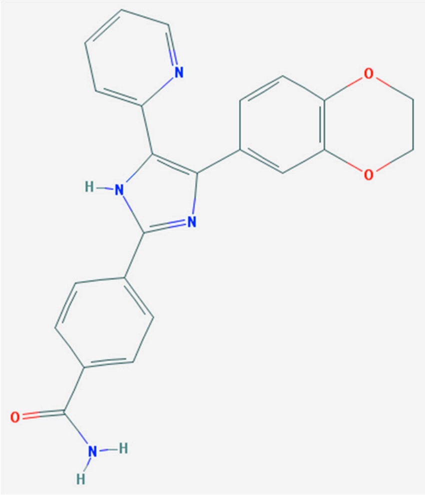	6419753
Casein Kinase II Inhibitor III, TBCA	934358-00-6	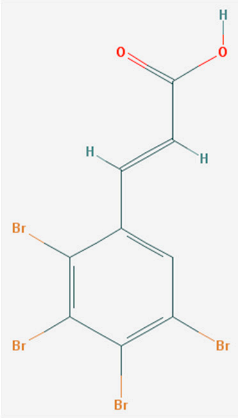	16760346
Cdk4 Inhibitor	546102-60-7	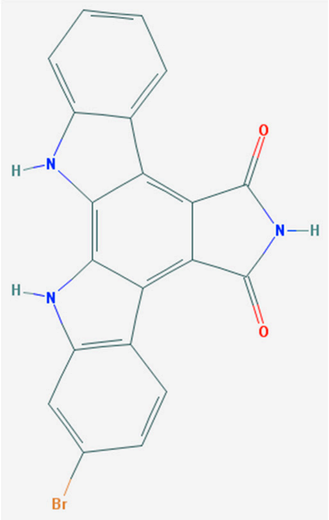	5330797

Table S1. *Cont.*

Description	CAS Number	Structure	PubChem Compound ID
Cdk4 Inhibitor II, NSC 625987	141992-47-4	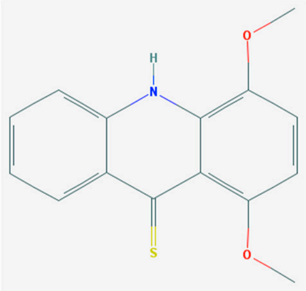	3004085
Cdk4 Inhibitor III	265312-55-8	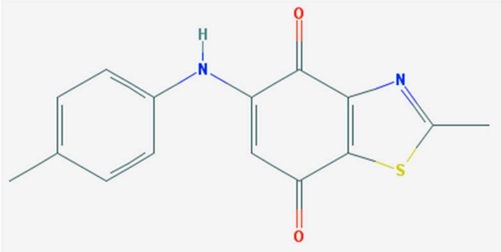	481747
Cdc2-Like Kinase Inhibitor, TG003	300801-52-9	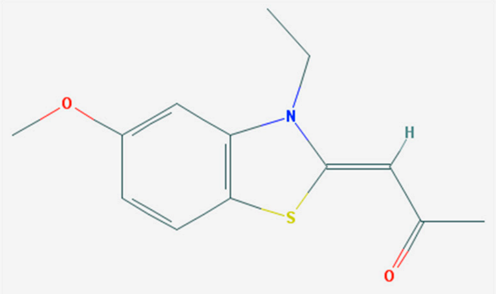	1893668
Chk2 Inhibitor II	516480-79-8	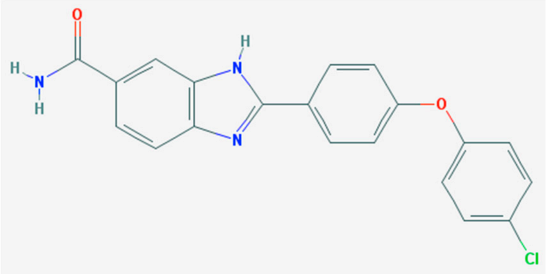	9969021
Compound 52	212779-48-1	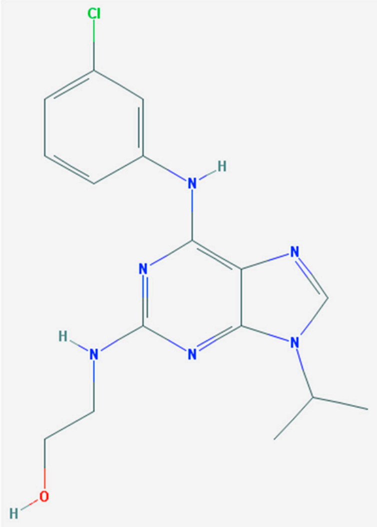	2856

Table S1. *Cont.*

Description	CAS Number	Structure	PubChem Compound ID
**Cdk2 Inhibitor III**	**199986-75-9**	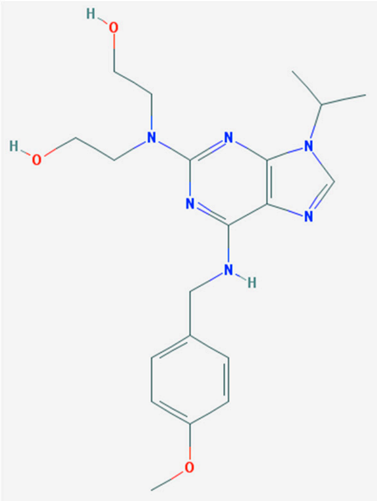	**6918386**
**Cdk2 Inhibitor IV, NU6140**	**444723-13-1**	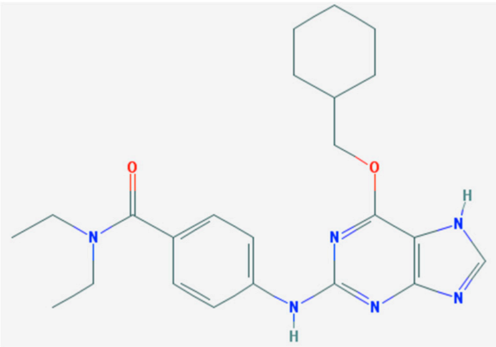	**10202471**
**Cdk/Crk Inhibitor**	**784211-09-2**	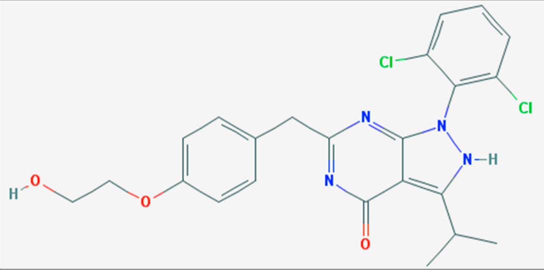	**9549301**
**ERK Inhibitor III**		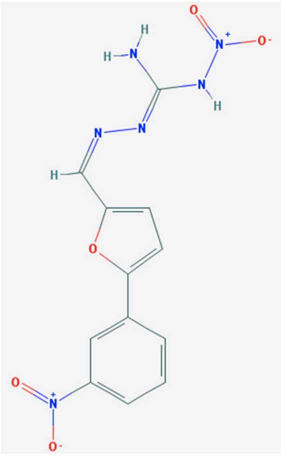	**5339183**

Table S1. *Cont.*

Description	CAS Number	Structure	PubChem Compound ID
ROCK Inhibitor, Y-27632	146986-50-7	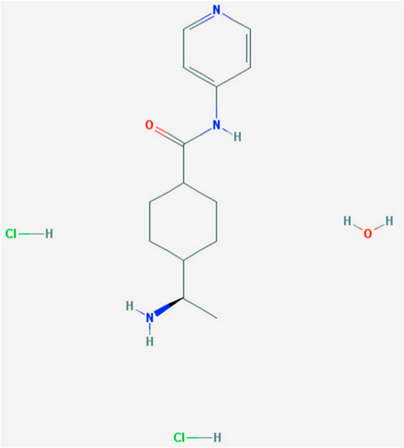	9797929
ERK Inhibitor II, FR180204	865362-74-9	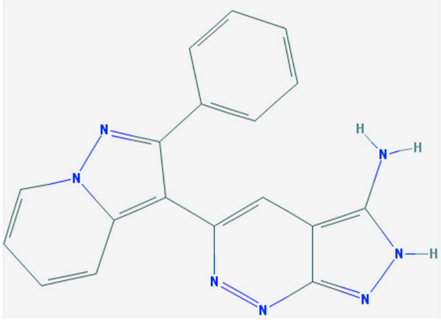	11493598
ERK Inhibitor II, Negative control		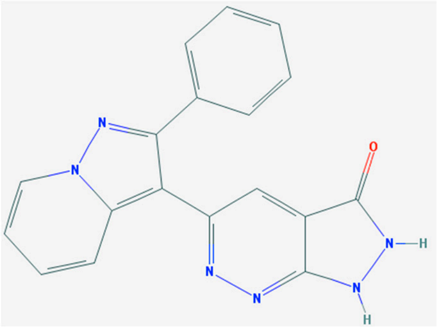	16760417
Fascaplysin, Synthetic	114719-57-2	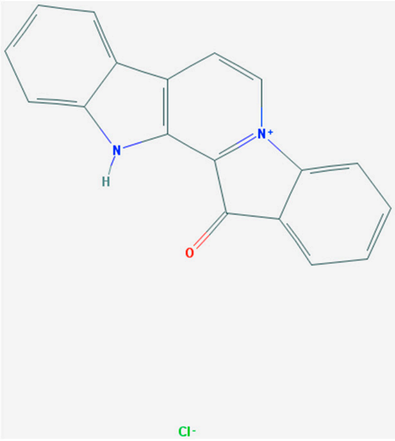	73292

Table S1. *Cont.*

Description	CAS Number	Structure	PubChem Compound ID
GSK-3b Inhibitor I	327036-89-5	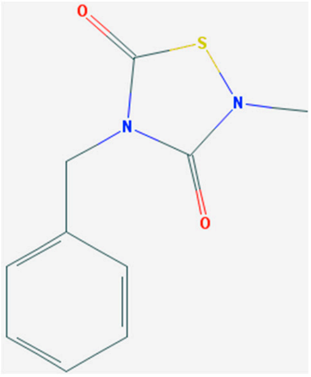	4124851
GSK-3b Inhibitor II	478482-75-6	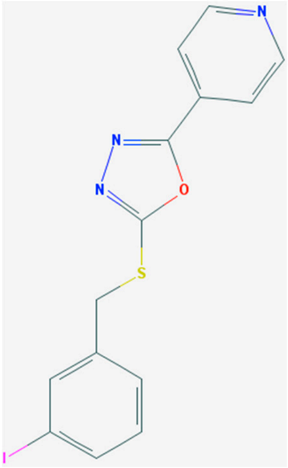	6539732
GSK-3b Inhibitor VIII	487021-52-3	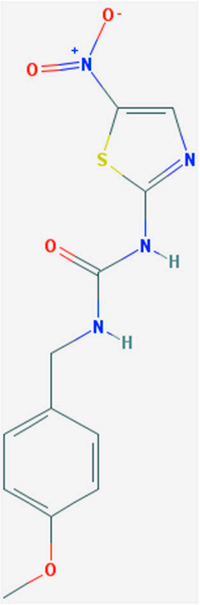	448014

Table S1. *Cont.*

Description	CAS Number	Structure	PubChem Compound ID
**GSK-3 Inhibitor IX**	**667463-62-9**	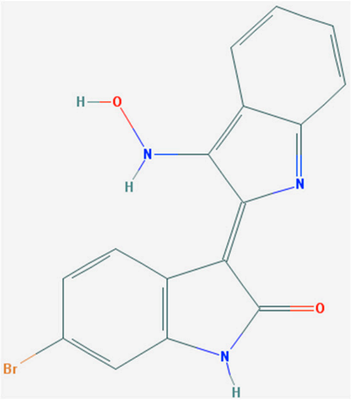	**5287844**
**GSK-3 Inhibitor X**	**667463-85-6**	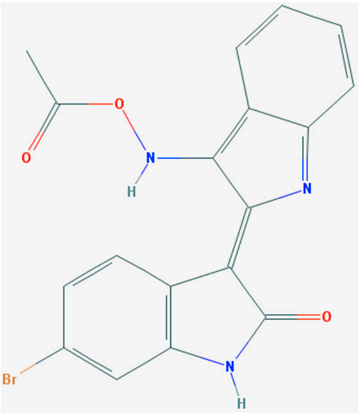	**6538818**
**GSK-3b Inhibitor XI**	**626604-39-5**	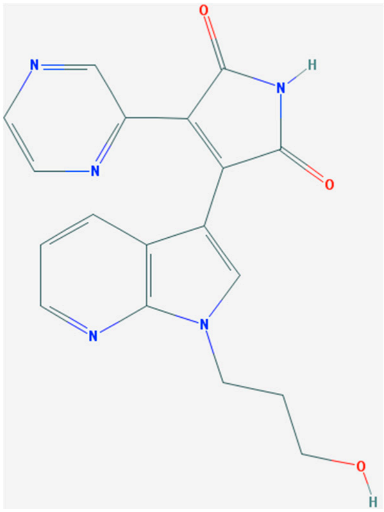	**10020713**
**SU6656**	**330161-87-0**	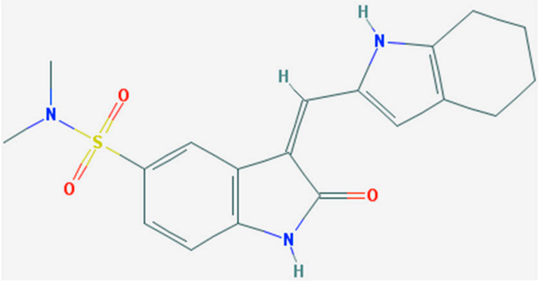	**5312137**

Table S1. *Cont.*

Description	CAS Number	Structure	PubChem Compound ID
GSK-3 Inhibitor XIII	404828-08-6	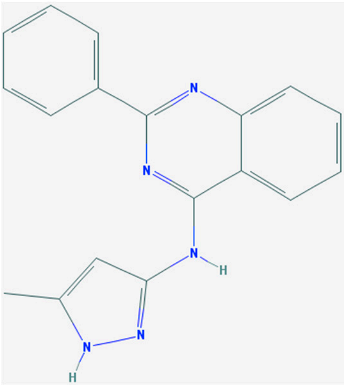	6419766
Isogranulatimide	244148-46-7	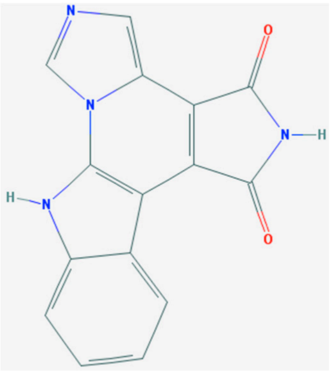	6419741
IC261	186611-52-9	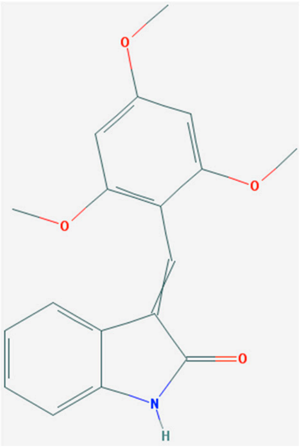	3674
IKK-2 Inhibitor IV	507475-17-4	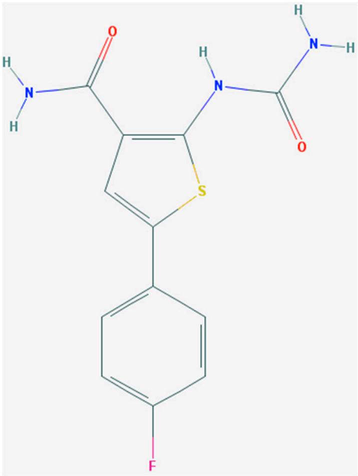	9903786

Table S1. *Cont.*

Description	CAS Number	Structure	PubChem Compound ID
Indirubin Derivative E804		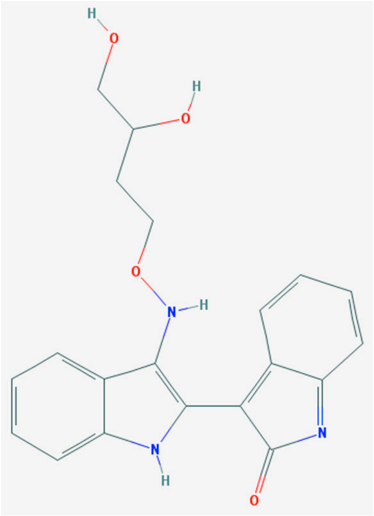	6419764
JNK Inhibitor II	129-56-6	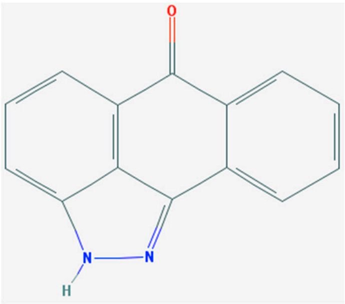	8515
JNK Inhibitor, Negative Control	54642-23-8	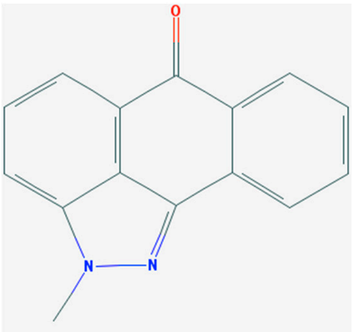	11665831
JNK Inhibitor V	345987-15-7	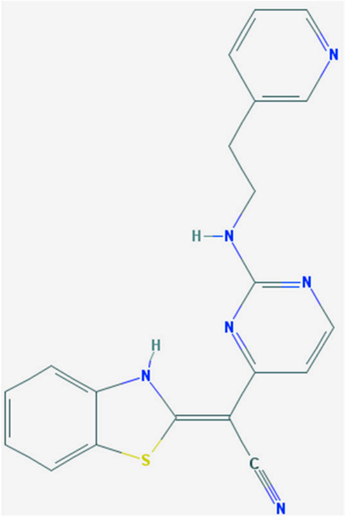	11422035

Table S1. *Cont.*

Description	CAS Number	Structure	PubChem Compound ID
JNK Inhibitor IX	312917-14-9	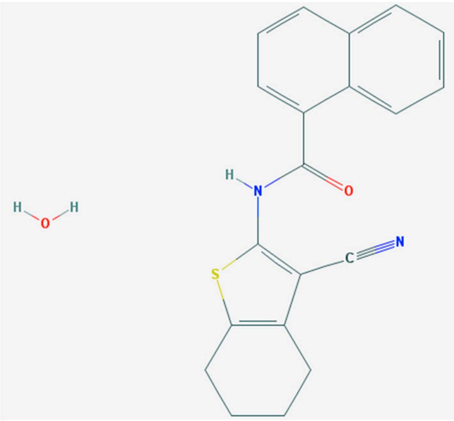	16760525
MK2a Inhibitor	41179-33-3	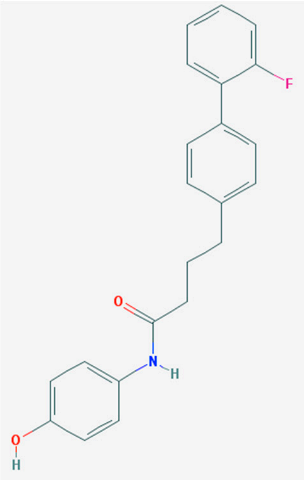	11382492
JNK Inhibitor VIII	894804-07-0	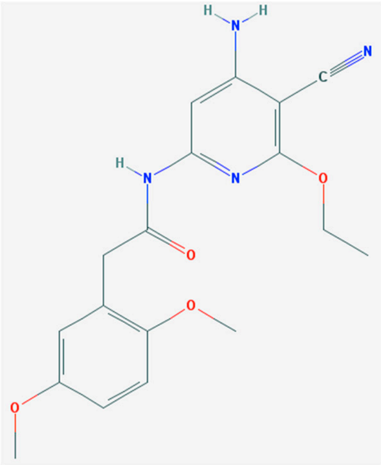	11624601
K-252a, Nocardiopsis sp.	97161-97-2	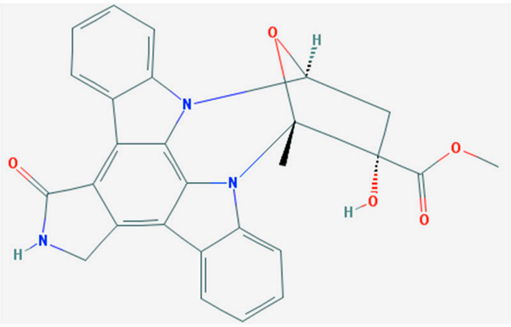	490561

Table S1. *Cont.*

Description	CAS Number	Structure	PubChem Compound ID
Kenpaullone	142273-20-9	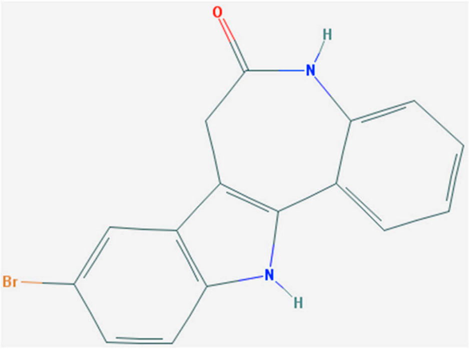	3820
KN-93	139298-40-1	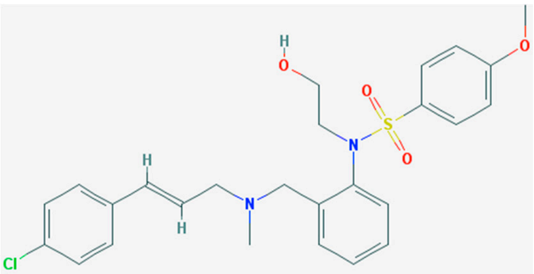	5312122
MEK Inhibitor I		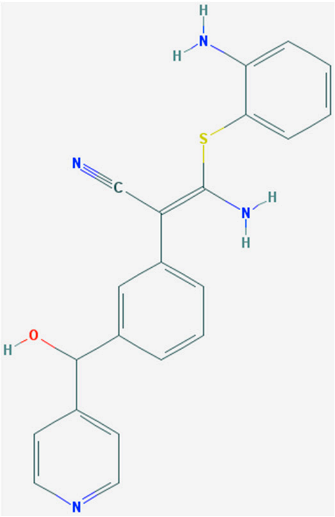	9951490
MEK Inhibitor II	623163-52-0	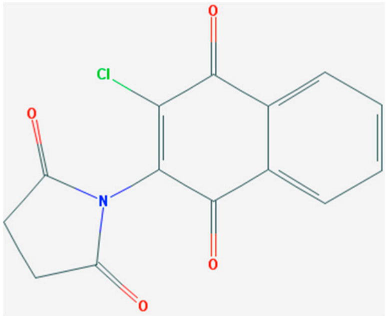	389898

Table S1. *Cont.*

Description	CAS Number	Structure	PubChem Compound ID
MEK1/2 Inhibitor	305350-87-2	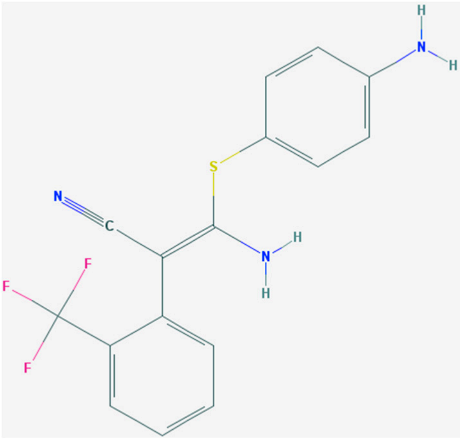	9549284
MNK1 Inhibitor	522629-08-9	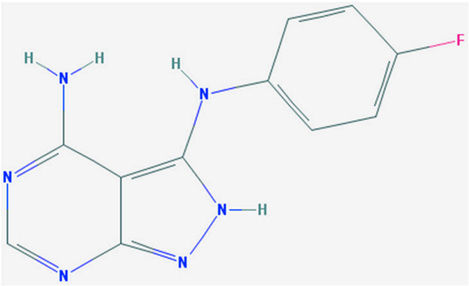	11644425
NF-kB Activation Inhibitor	545380-34-5	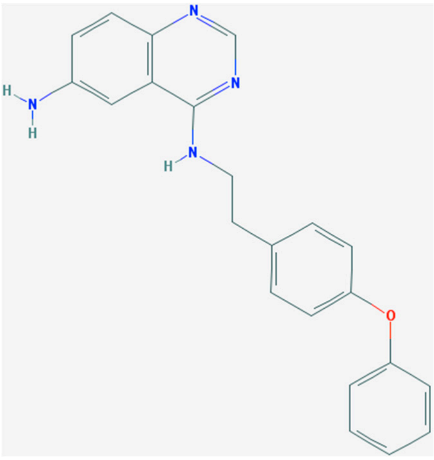	509554
p38 MAP Kinase Inhibitor III	581098-48-8	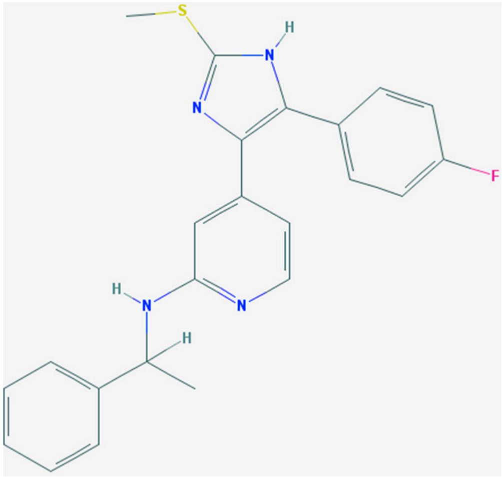	6419739

Table S1. *Cont.*

Description	CAS Number	Structure	PubChem Compound ID
p38 MAP Kinase Inhibitor	219138-24-6	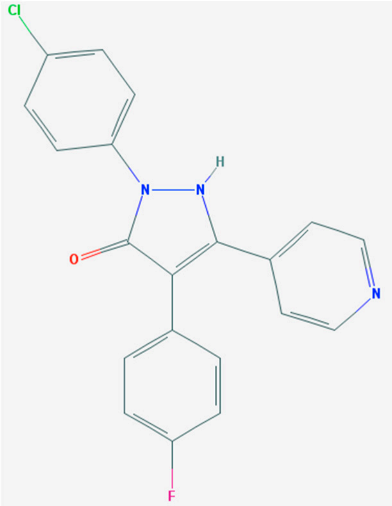	4665
PD 98059	167869-21-8	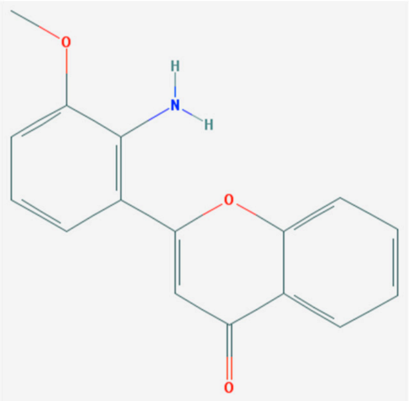	4713
PD 169316	152121-53-4	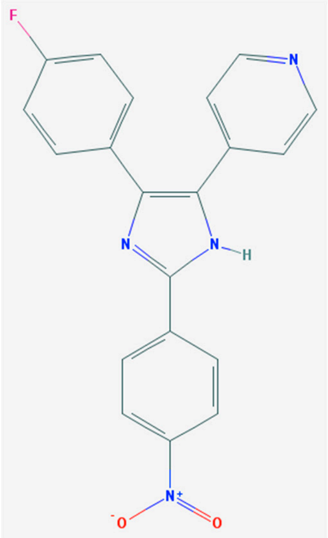	4712

Table S1. *Cont.*

Description	CAS Number	Structure	PubChem Compound ID
SB220025	165806-53-1	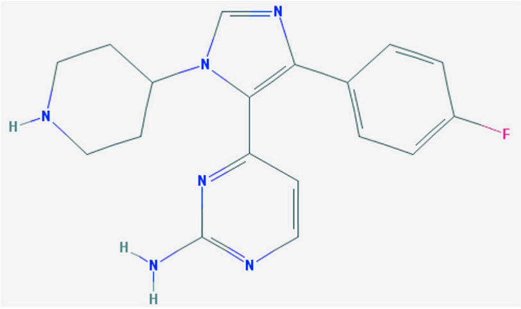	5164
Purvalanol A	212844-53-6	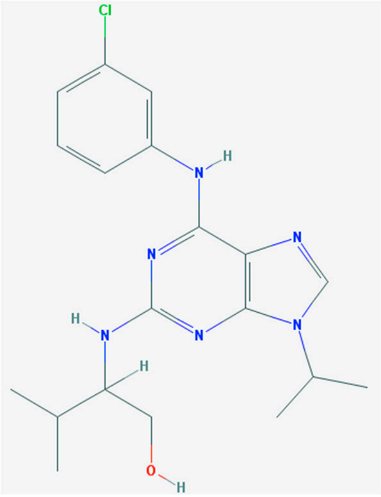	4987
GSK3b Inhibitor XII, TWS119	601514-19-6	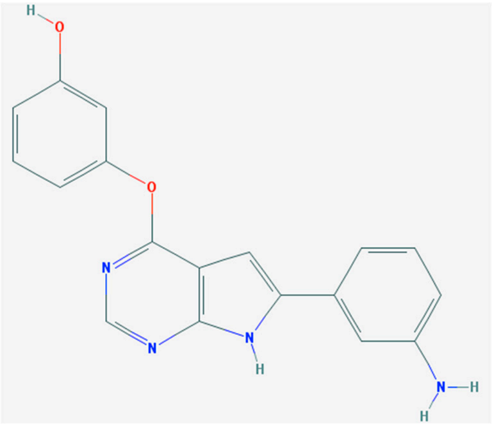	9549289
H-89, Dihydrochloride	127243-85-0	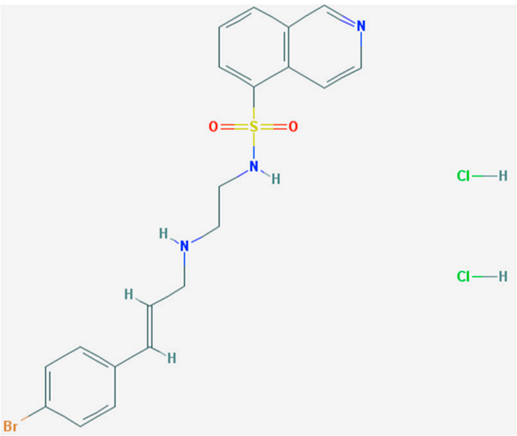	5702541

Table S1. *Cont.*

Description	CAS Number	Structure	PubChem Compound ID
SB 202474, Negative control for p38 MAPK inhibition studies	172747-50-1	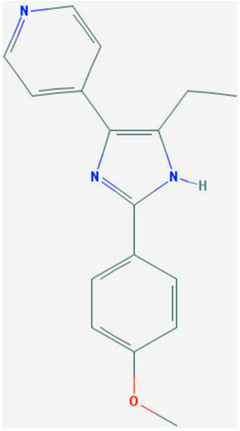	5162
SB 202190	152121-30-7	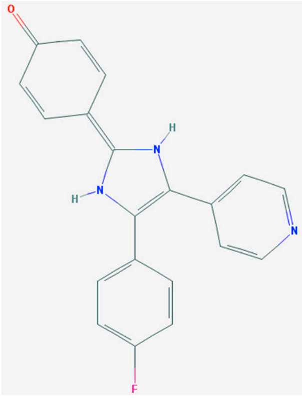	5353940
SB 203580	152121-47-6	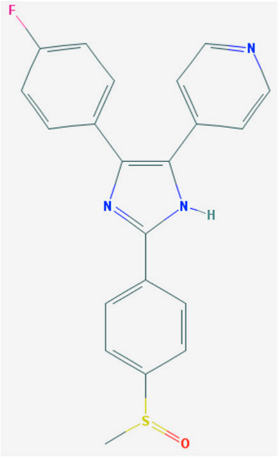	176155
HA 1077, Dihydrochloride Fasudil	103745-39-7	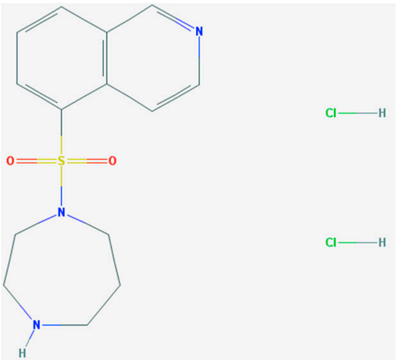	16219471

Table S1. *Cont.*

Description	CAS Number	Structure	PubChem Compound ID
**SB 218078**	**135897-06-2**	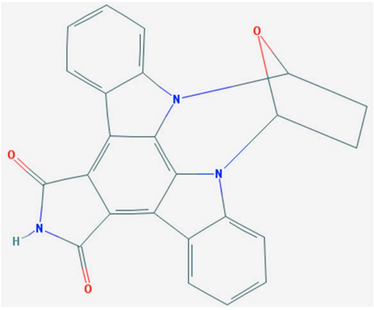	**3387354**
**SC-68376**	**318480-82-9**	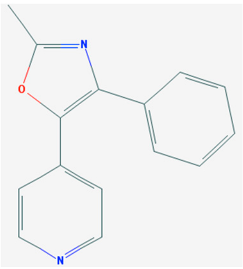	**5174**
**SKF-86002**	**72873-74-6**	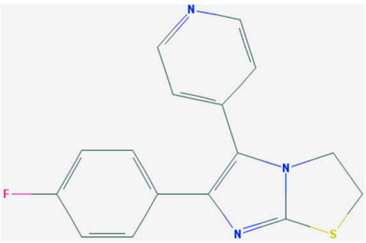	**5228**
**Sphingosine Kinase Inhibitor**	**1177741-83-1**	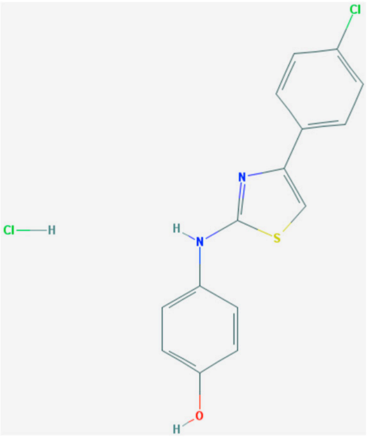	**16760659**
**Staurosporine, Streptomyces sp.**	**62996-74-1**	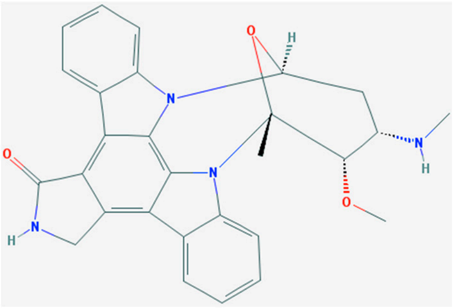	**451705**

Table S1. *Cont.*

Description	CAS Number	Structure	PubChem Compound ID
STO-609	52029-86-4	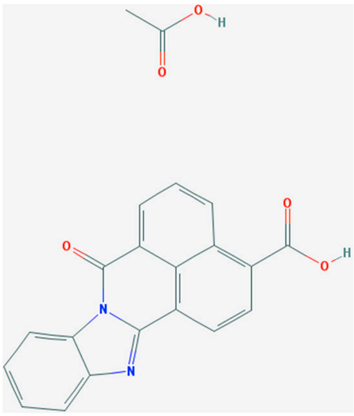	16760660
SU9516	666837-93-0	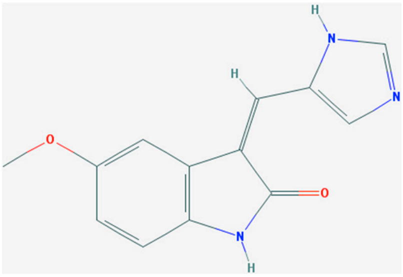	5289419
Tpl2 Kinase Inhibitor	871307-18-5	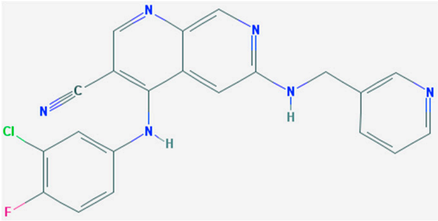	9549300
AMP	61-19-8	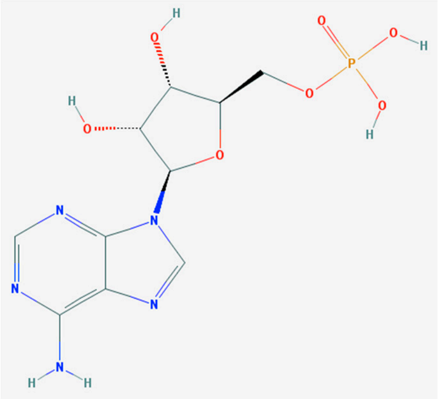	6083
ADP	58-64-0	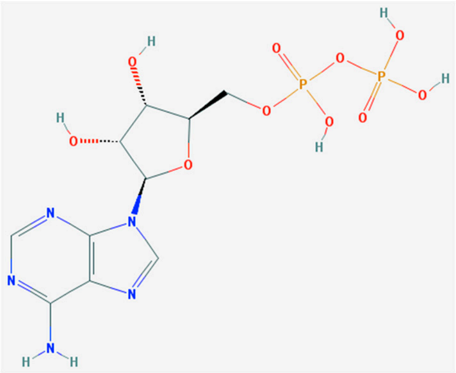	6022

Table S1. *Cont.*

Description	CAS Number	Structure	PubChem Compound ID
**ATP**	**56-65-5**	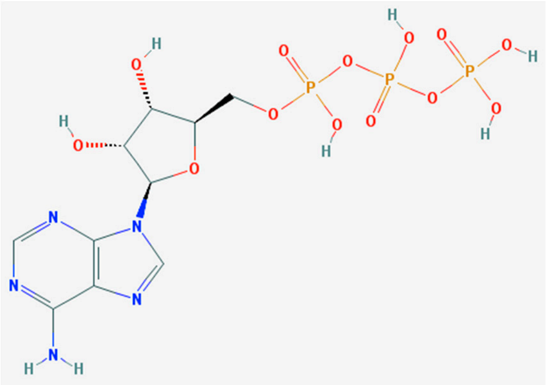	**5957**
**ATP-gammaS**	**35094-46-3**	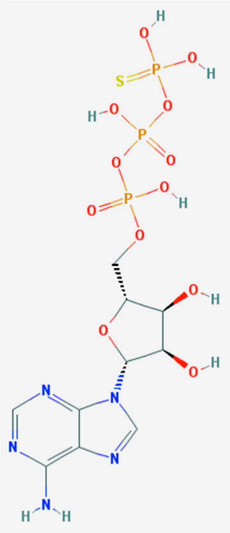	**440317**
**SU11652**		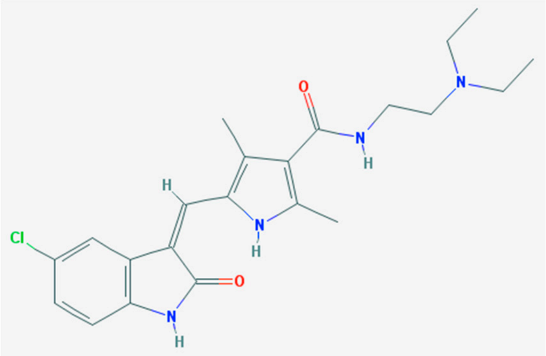	**5329103**

References

1. Bolton, E.; Wang, Y.; Thiessen, P.A.; Bryant, S.H. Chapter 12—PubChem: Integrated Platform of Small Molecules and Biological Activities. *Annu. Rep. Comput. Chem.* **2008**, *4*, 217–241.
